# Involuntary Attention in Healthy Older Adults at Electroencephalographic Risk of Cognitive Decline: An ERP Study

**DOI:** 10.1002/brb3.70970

**Published:** 2025-10-20

**Authors:** Mauricio González‐López, Rodolfo Solís‐Vivanco, Thalía Harmony, Thalía Fernández

**Affiliations:** ^1^ Laboratorio de Psicofisiología, Departamento de Neurobiología Conductual y Cognitiva, Instituto de Neurobiología Universidad Nacional Autónoma de México Juriquilla Queretaro Mexico; ^2^ Escuela Nacional de Estudios Superiores Juriquilla Universidad Nacional Autónoma de México Juriquilla Queretaro Mexico; ^3^ Escuela de Psicología Universidad Anáhuac Querétaro El Marqués Queretaro Mexico; ^4^ Laboratorio de Neurofisiología Clínica y Cognitiva Instituto Nacional de Neurología y Neurocirugía Manuel Velasco Suárez Ciudad de México Mexico; ^5^ Facultad de Psicología Universidad Nacional Autónoma de México Ciudad de México Mexico; ^6^ Unidad de Investigación en Neurodesarrollo, Departamento de Neurobiología Conductual y Cognitiva, Instituto de Neurobiología Universidad Nacional Autónoma de México Juriquilla Queretaro Mexico

**Keywords:** cognitive aging, cognitive decline, event‐related potentials (ERPs), involuntary attention, quantitative electroencephalography (qEEG)

## Abstract

**Background:**

The aging of the global population underscores the urgent need for the validation of biomarkers that can reliably distinguish individuals at risk of neurocognitive disorders. While quantitative EEG (qEEG) studies suggest that excessive theta activity predicts cognitive decline in the long term, less is known about whether increased theta can index current reduced cognitive resources, including attention, in older adults.

**Methods:**

This study compared the distraction event‐related potential (Mismatch Negativity (MMN)‐P3a‐Reorientation Negativity (RON)), linked to involuntary attention, between 25 older adults with excessive theta activity (risk group, RG) and 25 controls (CG). Participants underwent an auditory duration discrimination task with standard and deviant tones while an EEG was recorded.

**Results:**

Behaviorally, both groups showed distraction effects, with no significant differences between them. There were no significant differences in the amplitudes of the distraction potential between groups. However, the RG exhibited delayed P3a latencies at midline centroparietal regions and delayed RON at left parietal regions. Topographically, the RG displayed a bilateral RON effect (vs. the CG's right‐lateralized distribution). The MMN latency remained unaffected.

**Conclusions:**

These findings suggest delayed attentional orientation (P3a) and reorientation (RON) in at‐risk adults, despite preserved behavioral performance. The atypical RON distribution may reflect compensatory mechanisms mitigating cognitive inefficiencies. While at‐risk‐related neural delays did not yet manifest behaviorally, they highlight early electrophysiological markers of subclinical attentional decline. This underscores the utility of increased theta activity in detecting preclinical alterations, challenging the reliance on neuropsychological tests alone.

## Graphical abstract

This study compared the distraction event‐related potential between 25 older adults with excessive theta activity (risk group, RG) and 25 controls (CG). Participants underwent an auditory duration discrimination task with standard and deviant tones while an EEG was recorded to obtain ERPs. Behaviorally, both groups showed distraction effects, with no significant differences between them. There were no significant differences in the amplitudes of the distraction potential between groups. However, the RG exhibited delayed P3a latencies at midline centroparietal regions and delayed RON at left parietal regions. These findings suggest delayed attentional orientation (P3a) and reorientation (RON) in at‐risk adults, despite preserved behavioral performance.

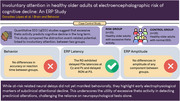

## 1 Introduction

Life expectancy has exhibited a significant rise worldwide over the last decades, and most countries have witnessed a shift in their population distributions towards older ages, a phenomenon that has been termed population aging. According to the World Health Organization (2022), by 2030, one‐sixth of the world population will be over 60 years old, and the number of persons over 80 will triple between 2020 and 2050. Moreover, aging is the leading risk factor for the development of a neurocognitive disorder (NCD), and the overall prevalence of NCDs may be as high as 30% in people aged 85 or older (American Psychiatric Association [APA], 2022).

Due to the potential of early interventions to delay the onset of clinical features of NCDs, the search for reliable biomarkers that can detect people at risk or in the preclinical phases of these disorders has become mandatory. One neurobiological technique that can provide potential biomarkers for NCD is quantitative electroencephalography (qEEG), which has shown promissory results. In particular, the excess of theta activity (4–8 Hz) has shown usefulness as a predictor of future cognitive decline in healthy elderly individuals (Prichep et al. 2006; van der Hiele et al. 2008) and the progression to major NCD in patients with current mild NCD (Moretti et al. 2009, 2011; Rossini et al. 2006). This has also been supported by Musaeus et al. (2018), who reported a positive correlation between theta activity and cognitive decline in nearly 400 subjects. Additional research has found a relationship between theta activity in the qEEG and cerebrospinal fluid biomarkers of Alzheimer's disease, that is, beta‐amyloid (Spinelli et al. 2022), a finding that has been shown in both healthy participants (Stomrud et al. 2010) and those with a diagnosed NCD (Hata et al. 2017).

In the same line, our research group has observed differences in cortical thickness using MRI between a group of healthy older adults with excessive theta activity and a control group of healthy older adults with normal EEG, where the former exhibited larger cortical thickness in the right temporal areas (Castro‐Chavira et al. 2016) and alterations in functional and effective connectivity within the nodes of the default mode network (González‐López et al. 2022). Noticeably, there were no cognitive differences between these groups when measured using behavioral and neuropsychological tests. This lack of behavioral differences was later confirmed by employing highly demanding cognitive tasks in other samples (Sigg‐Alonso et al. 2025).

The neurocognitive assessment of an individual is typically carried out using neuropsychological batteries that have been standardized for a specific population, allowing for the establishment of a normality criterion by comparing the individual's score to a normative database. However, it is important to note that behavioral scores, while widely used, do not consider the neural processes that lead to a particular behavioral response. This limitation underscores the need for a more comprehensive approach, such as the use of electrophysiological tools like event‐related potentials (ERPs), which can track the sequence of neural responses that precede a motor action with high temporal resolution. Our research group has previously reported that subjects with excessive theta activity exhibit atypical patterns in ERPs associated with syntactic processing (Alatorre‐Cruz et al. 2019) and inhibitory control (Sánchez‐Moguel et al. 2018). Since we have identified differences in brain processing that involve complex cognitive functions, such as executive functions and syntactic processing, here we explore a fundamental cognitive process: attention.

Attention allows for relevant stimuli to be selected, and it undergoes some deterioration during aging, affecting both its top‐down (i.e., voluntary attention) and bottom‐up (i.e., involuntary attention) subcomponents. In the case of involuntary attention, ERP research has shown changes in older participants as revealed by the so‐called distraction potential, composed of the Mismatch Negativity (MMN), the P3a, and the Reorientation Negativity (RON), each one respectively associated with the automatic monitoring of the context through sensory processes and detection of changes in the environment, the orientation of attentional resources towards the detected change, and the reorientation of these resources back to the original task (Schroger and Wolff 1998; Solís‐Vivanco et al. 2011).

Horváth et al. (2009) reported that aging influences the ability to reorient attention when presented with a nonrelevant distractor during a duration discrimination task (DDT), as revealed by longer latencies of the P3a and RON components in older adults compared to young adults. Using the same task, Getzmann et al. (2013) compared a group of young adults with two groups of older adults: one with good performance and one with poor performance. They observed smaller amplitudes of the MMN in both groups of older adults, especially in the poor‐performance group. This suggests that the automatic detection of deviant stimulus properties is less efficient in aging and may result in the need to use more controlled cognitive strategies (as opposed to automatic processes) to achieve a good level of performance in the task. Additionally, the high‐performing older adult group showed smaller amplitudes of the P3a and RON components than the poor‐performing older adult group, suggesting that the better‐performing group experienced fewer distraction effects and, therefore, less need for resources to reorient attention to task‐relevant characteristics. The study concluded that, in general, poorer‐performing older adults need to utilize more cognitive resources to compensate for increased distractibility.

While those studies provided relevant information about the neurophysiological correlates of involuntary attention in older adults, there is a lack of knowledge about whether they can be affected in adults with an electroencephalographic risk of cognitive decline. Therefore, our objective was to compare the distraction potential between adults with and without excessive theta activity to further characterize this population in neurocognitive terms. We hypothesized that the at‐risk group (RG) would exhibit a more prominent distraction effect as revealed by task performance and increased amplitudes and latencies of the corresponding ERP components.

## 2. Method

This study was approved by the Bioethics Committee of the Institute of Neurobiology of the National Autonomous University of Mexico (INEU/SA/CB/109‐HRM30) and was conducted in accordance with the Declaration of Helsinki.

### 1 Participants

An a priori sample size calculation was conducted using G*Power (version 3.1.9.6) for a repeated‐measures ANOVA with a 2 (condition) × 2 (group) mixed design, focusing on the interaction effect. Based on the differences observed by previous research of our group (Sánchez‐Moguel et al. 2018), a medium‐to‐large effect size was assumed for this study. Specifically, *f* = 0.3 was used for the power analysis. With an alpha level of 0.05 and desired power (1 − β) of 0.80, the analysis indicated that a total sample size of 24 would be required to detect a statistically significant interaction between group and time. Regarding the detection of the group effect, a minimum sample size of 40 would be required.

Participants were recruited from the general population through word‐of‐mouth recommendations and radio and online advertisements. Inclusion criteria included being at least 55 years old, right‐handedness, and a minimum of 9 years of formal education. All participants had an IQ above 80, as revealed by the Wechsler Adult Intelligence Scale, Fourth Edition (WAIS‐IV). Additionally, blood analyses revealed healthy ranges for glucose, cholesterol, triglycerides, hemoglobin, and thyroid‐stimulating hormone in all of them. Participants with a history of neurological diseases or a current diagnosis of any psychiatric disorder were excluded from the study. Educational level was measured by asking participants to report the total number of years of formal education completed. Socioeconomic status was assessed by collecting self‐reported total household income and the number of individuals dependent on that income, from which we calculated the household per capita income. Additionally, participants were asked to self‐rate their socioeconomic position on a 10‐point scale considering income, education, occupation, and access to resources.

After we verified the fulfillment of the inclusion criteria, an EEG was recorded (see below). All participants with abnormal EEG brain wave patterns, as revealed by visual inspection, were excluded from the study. This decision was made to maintain the internal validity and focus of the study, as our primary aim was to isolate the effect of excess theta activity as a potential marker of neurocognitive risk. Including participants with other EEG abnormalities would have increased heterogeneity in the sample and potentially confounded the interpretation of the results. Those participants with an excess of theta absolute power (*z* > 1.96) in at least one channel according to norms (Bosch et al. 2020) were assigned to the at‐RG (*n* = 25). Individuals with a normal EEG, that is, those with all frequency bands within the range of −1.96 to 1.96 *z* values, were assigned to the control group (CG; *n* = 25). Thus, a total of 50 participants met the inclusion criteria for this study (mean age = 66.03; standard deviation = 6.89; 33 women, 17 men, and zero nonbinary individuals). All participants signed a written informed consent form before any experimental procedure was done. The groups did not differ in terms of gender, age, years of education, or IQ (see Table 1).

**TABLE 1:** Demographic and psychometric assessments of intelligence of the control group (CG) and the risk group (RG) according to the WAIS‐IV.

	CTRL GROUP (*n* = 31)		RISK GROUP (*n* = 37)		TEST	*p*
Gender	16 female, 9 male		17 female, 8 male		𝝌^2^ = 0.0891	0.76
	Mean	SD	Mean	SD		
Age	65.76	7.06	65.68	7.76	*t* = 0.038	0.96
Years of education	19.80	4.67	19.21	4.24	*t* = 0.463	0.64
VCI	122.08	9.70	122.04	11.62	*t* = 0.013	0.98
PRI	107.80	12.34	111.88	12.42	*t* = 1.165	0.24
WMI	109.04	9.12	109.16	11.35	*t* = 0.041	0.96
PSI	109.36	8.24	112.48	8.10	*t* = 1.349	0.18
FSIQ	114.40	9.70	116.60	11.28	*t* = 0.739	0.46

Abbreviations: FSIQ, full‐scale IQ; PRI, perceptual reasoning index; PSI, processing speed index; VCI, verbal comprehension index; WMI, working memory index.

### 2 Resting EEG Recording

A 10 min resting EEG was recorded in participants with their eyes closed using a MEDICID IV system (Neuronic Mexicana, SA, Mexico) and TrackWalker version 2.0 software, with 19 Ag/AgCl electrodes mounted on an elastic cap (Electro‐Cap, International Inc., Eaton, Ohio, USA) according to the International 10–20 System and referenced to linked earlobes. The participants were seated in comfortable chairs in a faradized, soundproofed, air‐conditioned, and dimly lit room. The EEG was digitized at a sampling rate of 200 Hz and amplified with a gain of 20,000. The impedances of the electrodes were maintained below 5 kΩ. None of the participants were under the effects of any psychotropic drug. During the recording, they were instructed not to fall asleep.

Following the procedure described by Bosch et al. (2020) to obtain the norms, 24 artifact‐free epochs of 2.56 s each were visually selected from each recording by an expert in electroencephalography. The selected segments throughout the recording had to maintain the frequency and amplitude characteristics of the posterior dominant rhythm, thereby excluding any activity associated with drowsiness. The qEEG analysis was performed offline using a fast Fourier transform, and cross‐spectral matrices were calculated with a frequency resolution of 0.3906 Hz. Absolute and relative power values were obtained for each frequency band. The geometric power was subtracted from each cross‐spectrum. This correction involved rescaling the power spectrum to reduce variance unrelated to brain factors, resulting in a reduction of up to 42% (Hernández et al. 1994). Then, *z*‐scores for each measure of absolute power (z‐AP) were obtained by comparing the raw data to a normative database (Bosch et al. 2020), ensuring the validity of the results.

### 3 Experimental Task and Procedure

**FIGURE 1:**
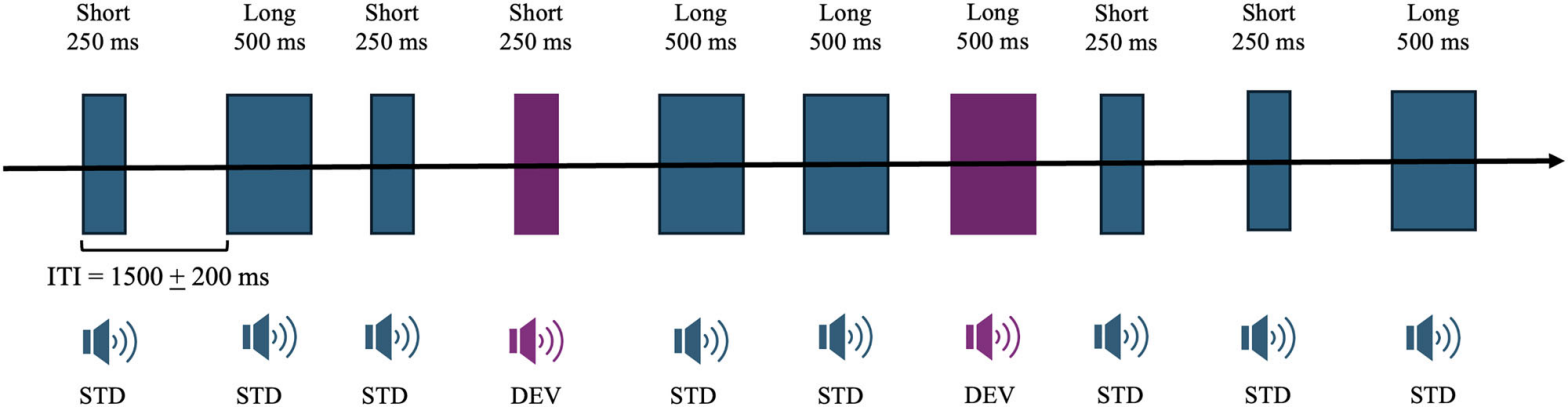
Schematic representation of the task. Each block represents a stimulus (short or long, which is relevant to the subject's task). For the analysis of the ERPs, two conditions are considered: standard (ST, blue blocks) or deviant (DV, purple blocks).

After the participants were assigned to one of the groups, they were scheduled to attend the lab to record the ERPs. Each participant completed a DDT (Figure 1), which has been widely used to study involuntary attention (e.g., Schroger and Wolff 1998; Solís‐Vivanco et al. 2011). The task was programmed using Stim2 software (Compumedics NeuroScan, USA). A total of 800 stimuli were binaurally presented at approximately 80 dB. Half of the stimuli were short (250 ms), and the other half were long (500 ms). Additionally, 85% of the stimuli had a frequency of 1000 Hz (standard stimuli [ST], *n* = 640), and 15% had a frequency of 800 Hz or 1200 Hz (deviant stimuli [DV], 50% each, *n* = 160). The inter‐trial interval was randomized around 1500 ms with a range of ± 200 ms. Participants’ task was to respond as quickly as possible to the duration of the stimuli (short or long) using a response pad. During the task, the participants were seated on a comfortable chair in a faradayized, sound‐proofed, dimly lit room. Foam headphones connected to the NeuroScan equipment's stimulation box (Compumedics NeuroScan, USA) delivered the stimuli. Visual fixation was controlled during the task using a fixation cross located in the middle of the screen. Reaction times (RT) and accuracy were obtained for each participant.

### 4 ERP Recording and Processing

The EEG recording included 32 Ag/AgCl electrodes, positioned according to the International 10–10 System and attached to an elastic cap (Electro‐cap, International, Inc.). The data was recorded at a high 500 Hz sampling rate using the Neuroscan SynAmps 2 system (Compumedics NeuroScan, USA) with a 0.5–70 Hz bandwidth. To ensure accuracy, electrode impedances were kept below 5 kΩ. The EEG recordings were referenced online to the right earlobe (A2), and the left earlobe (A1) was recorded as a separate channel. Blinks and eye movements were monitored by two electrodes on the left eye's external supraorbital ridge and canthus.

The EEGs were subsequently imported into EEGLAB v2024.0 (Delorme and Makeig 2004) and ERPLAB v10.1 (Lopez‐Calderon and Luck 2014), open‐source MATLAB toolboxes. The EEGs were resampled to 250 Hz and filtered using a band‐pass IIR Butterworth filter with cutoff frequencies of 1 Hz and 30 Hz (24 dB/oct), replicating the settings of the original paper by Schröger and Wolff (1998), and then re‐referenced to the average of both earlobes. Artifact components, such as eye blinks, channel noise, and muscular or cardiac noise, were identified by independent component analysis (ICA) using the ICLabel toolbox for EEGLab (https://sccn.ucsd.edu/wiki/ICLabel) and removed from the EEG.

The epochs for computing the ERPs were defined by a window of −200 to 1000 ms relative to stimulus onset, and baseline correction was performed by subtracting the pre‐stimulus portion of the epoch (−200 to 0 ms). EEG epochs were further inspected for artifacts using three automatic artifact rejection procedures included in the ERPLab toolbox: (1) moving window peak‐to‐peak threshold (200 ms window width, 100 ms steps, 100 µV threshold), which scanned the epochs for abrupt voltage swings; (2) the step function (200 ms window width, 50 ms window step, 100 µV threshold), to exclude saccadic movements and similar artifacts; and (3) the blocking and flat line function to exclude epochs with amplifier saturation (598 ms duration with an amplitude tolerance of 2 µV). After artifact rejection, we retained an average of 394 trials (SD = 69) in the RG and 372 trials (SD = 105) in the CG for the standard condition, and an average of 110 trials (SD = 27) in the RG and 111 trials (SD = 35) in the CG for the deviant condition. Independent‐samples t‐tests revealed no significant differences between groups in the number of artifact‐free trials for either the standard condition (*t*_(48)_ = 0.85, *p* = 0.39) or the deviant condition (*t*_(48)_ = 0.12, *p* = 0.91), supporting the consistency and comparability of data quality across groups.

The epochs were averaged separately for each condition (ST and DV), obtaining the corresponding ERP for each condition for each individual. Finally, the difference potential for each participant was obtained by subtracting the potential associated with the ST stimuli from the potential associated with the DV stimuli (i.e., ERP[DV]—ERP[ST]).

The amplitude analysis was conducted by comparing the point‐by‐point voltage of the individual potentials for each group and stimulus. To determine the latency of each component, the highest negative (for the MMN and the RON) and positive (for the P3a) peaks were identified on the difference wave on nine electrodes (F3, Fz, F4, C3, Cz, C4, P3, Pz, P4) over the following windows: (1) MMN = 100–200 ms (negative area); (2) P3a = 270–480 ms (positive area); (3) RON = 500–700 (negative area). The windows were determined based on the point‐by‐point analysis of the effect of the total sample, that is, using the grand‐average ERPs where the two groups were collapsed. Lastly, mean amplitudes for each subject were calculated for the same time windows for each component to test for an interaction between the topographical distribution and the group.

### 5 Statistical Analysis

The statistical analysis of the behavioral data, conducted within subjects (i.e., the effect of the stimuli type), was carried out using a mixed ANOVA, with the group as the between‐subjects factor and the condition as the within‐subjects factor. The data for accuracy (percentage of correct answers) was transformed to ensure a normal distribution of the data using the arcsine transformation. The data of four participants were excluded from this analysis due to corruption of the data files.

The amplitude analyses for both within‐subject and between‐subject comparisons were conducted point‐by‐point using the Mass Univariate Toolbox (Groppe et al. 2011), which corrects for multiple comparisons while retaining a good degree of statistical power. To detect reliable differences between the ERPs to ST stimuli and DV stimuli, we conducted a paired, two‐tailed permutation test for each group based on the tmax statistic (Blair and Karniski 1993) using a family‐wise alpha level of 0.05. All time points between 0 and 900 ms at all 32 scalp electrodes were included in the test, and 2500 random within‐participant permutations of the data were used to estimate the distribution of the null hypothesis (i.e., no difference between conditions). Based on this estimate, critical *t*‐scores of ±4.73 (df = 24) were derived for the CG and of ±4.59 (df = 24) for the RG. To detect reliable differences between the groups, an independent‐sample two‐tailed permutation test based on the tmax statistic was used, with a family‐wise alpha level of 0.05. All time points between 100 and 800 ms at all 32 scalp electrodes were included in the test, and 2500 random between‐participant permutations of the data were used to estimate the distribution of the null hypothesis (i.e., no difference between groups). Based on this estimate, critical *t*‐scores of ±4.36 (df = 48) were derived.

The statistical analysis of the latencies was conducted using an independent, one‐tailed permutation test based on the tmax statistic (Blair and Karniski 1993), with a family‐wise alpha level of 0.05, as implemented in a MATLAB script developed by Groppe (2024). In this case, one‐tailed tests were employed because we formulated an a priori directional hypothesis. First, the higher risk of cognitive decline in the RG led us to expect longer latencies relative to controls. Second, a previous study from our group (Alatorre‐Cruz et al. 2019) reported increased latency in at‐risk participants. The latency values for the highest local peaks, as previously defined, were included in the test, and 5000 random permutations of the data were used to estimate the distribution of the null hypothesis (i.e., no difference between stimuli). Based on this estimate, critical *t*‐scores of −2.52, −2.37, and −2.45 (df = 48) were derived for the MMN, P3a, and RON, respectively.

Finally, a mixed ANOVA was conducted on nine electrodes (F3, Fz, F4, C3, Cz, C4, P3, Pz, P4) to test for the effects of an interaction between the topographical distribution and the group on the mean amplitude of the components. The group (CG vs. RG) was included as the between‐subject factor, while the two within‐subject factors were considered: (1) an anteroposterior factor (frontal, central, posterior) and (2) a laterality factor (left, midline, right). Statistics for this test are reported using the Greenhouse‐Geisser correction due to the lack of sphericity.

The permutation‐based mass univariate analysis utilized the publicly available script from Groppe et al. (2011), accessible at https://github.com/dmgroppe/Mass_Univariate_ERP_Toolbox. The permutation‐based *t*‐test script is publicly available at https://www.mathworks.com/matlabcentral/fileexchange/54585‐mult_comp_perm_t2‐data1‐data2‐n_perm‐tail‐alpha_level‐mu‐t_stat‐reports‐seed_state. ANOVAs were conducted using JASP software, which is freely available.

## 2 Results

### 2.1 Behavior

**FIGURE 2:**
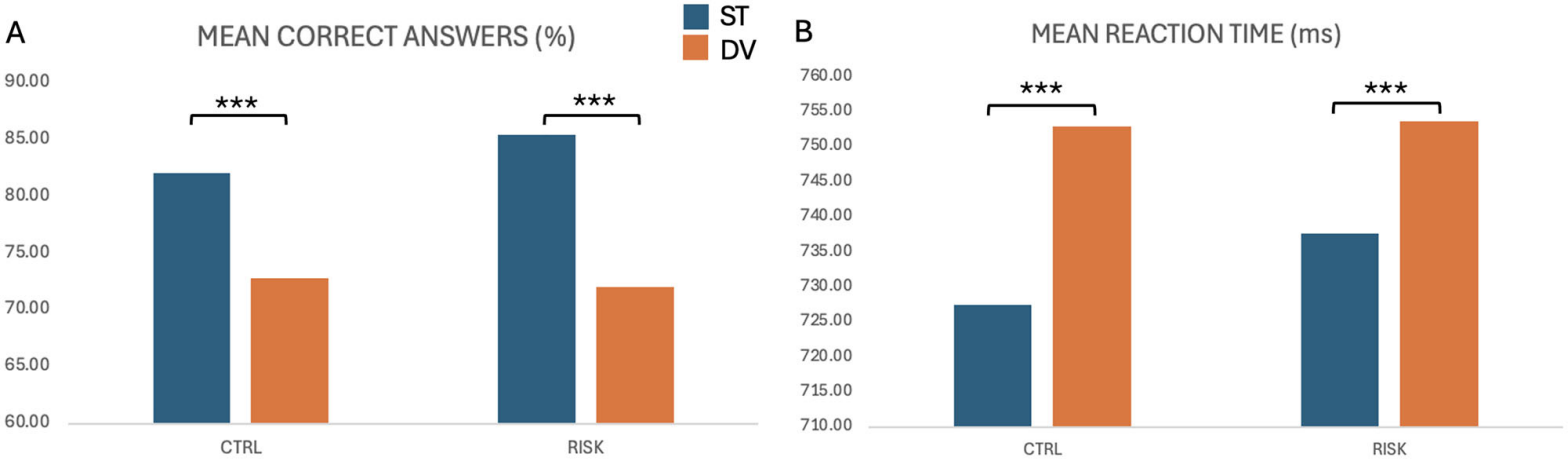
Means by group (CG vs. RG) of behavioral scores for (A) accuracy and (B) mean reaction time. ***p < 0.001.

Our analyses revealed a significant effect of the condition (ST vs. DV) on the behavioral measures. Participants exhibited a significantly better performance in terms of the percentage of correct responses for the ST stimuli (mean = 84.52 ± 12.89%) compared to the DV stimuli (mean = 72.96 ± 18.57%; *F*_(1,43)_ = 64.61, *p* < 0.0001, *η* ^2^ *_p_* = 0.6). Similarly, the ST stimuli led to shorter RTs (mean = 738.83 ± 60.8 ms) than the DEV condition (mean = 759.72 ± 73.65 ms; *F*_(1,43)_ = 12.02, *p* = 0.0012, *η* ^2^ *_p_* = 0.22). Notably, these effects were consistent across both groups, with neither a significant group effect nor an interaction between the factors observed in the accuracy rates or in the RT (Figure 2).

### 2.2 Event‐Related Potentials

**FIGURE 3:**
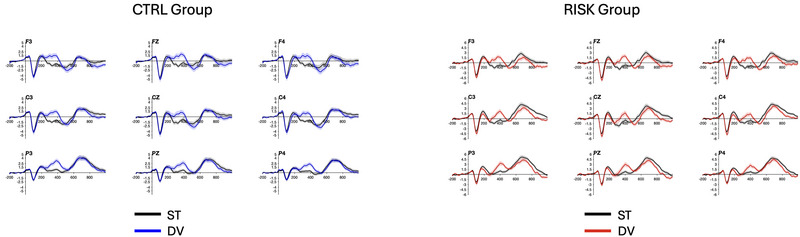
ERPs for each condition of the Control (left) and the Risk group (right) for each condition: standard (ST) and deviant (DV).

**FIGURE 4:**
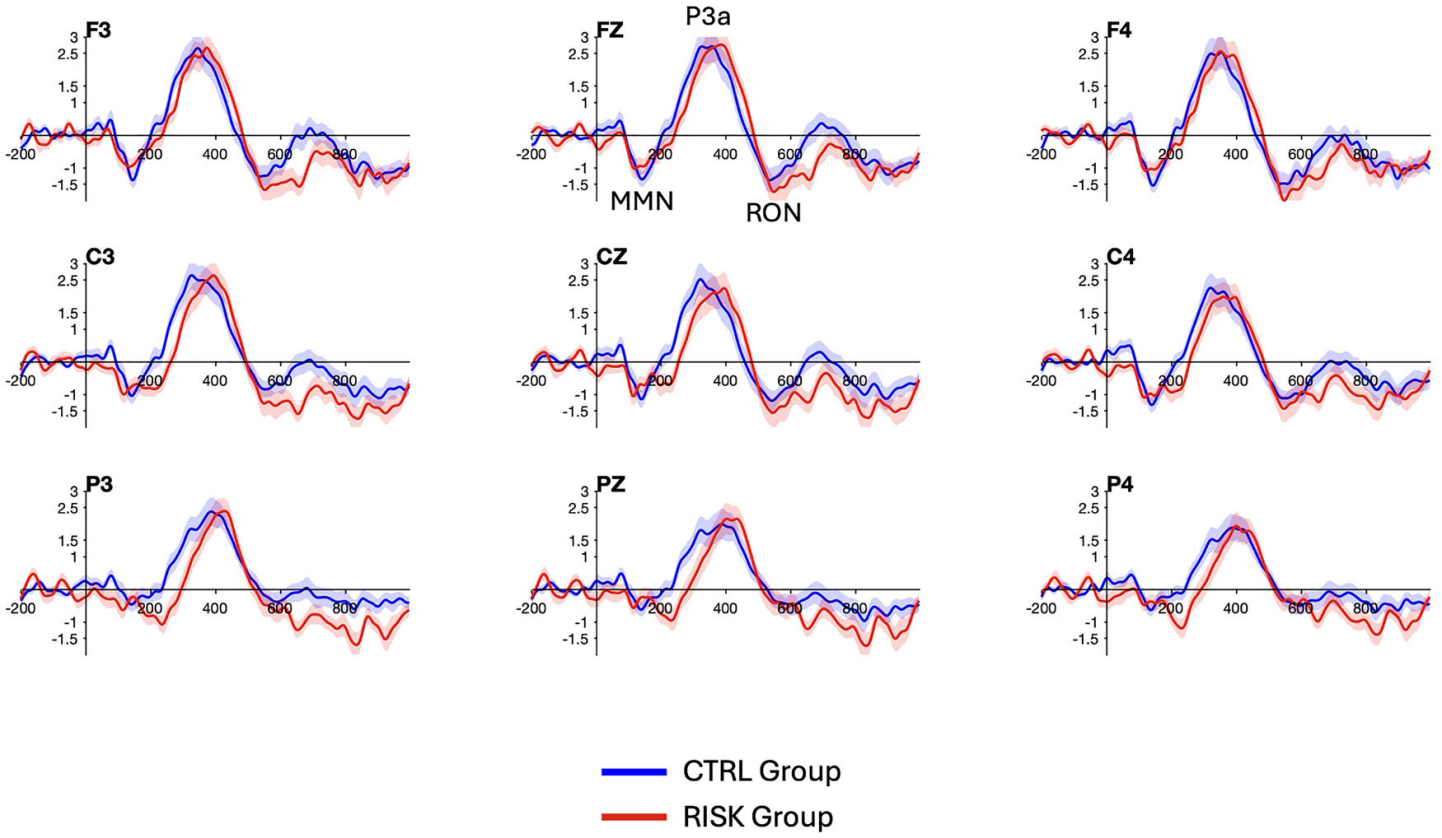
Differences in waves for the CTRL group (in blue) and the RISK group (in red). The three components of interest are labeled MMN, P3a, and RON in Pz.

Figure 3 shows the ERPs for each stimulus type and group, and Figure 4 shows the difference waves for each group.

#### 2.2.1 Between‐Subjects Amplitude Comparison

There were no significant differences across the defined epochs in the distraction potential (i.e., the difference wave) between the groups. In other words, no time points exceeded the critical *t* value established for the comparison between the RG and CG.

#### 2.2.2 Within‐Subjects Amplitude Comparison

**FIGURE 5:**
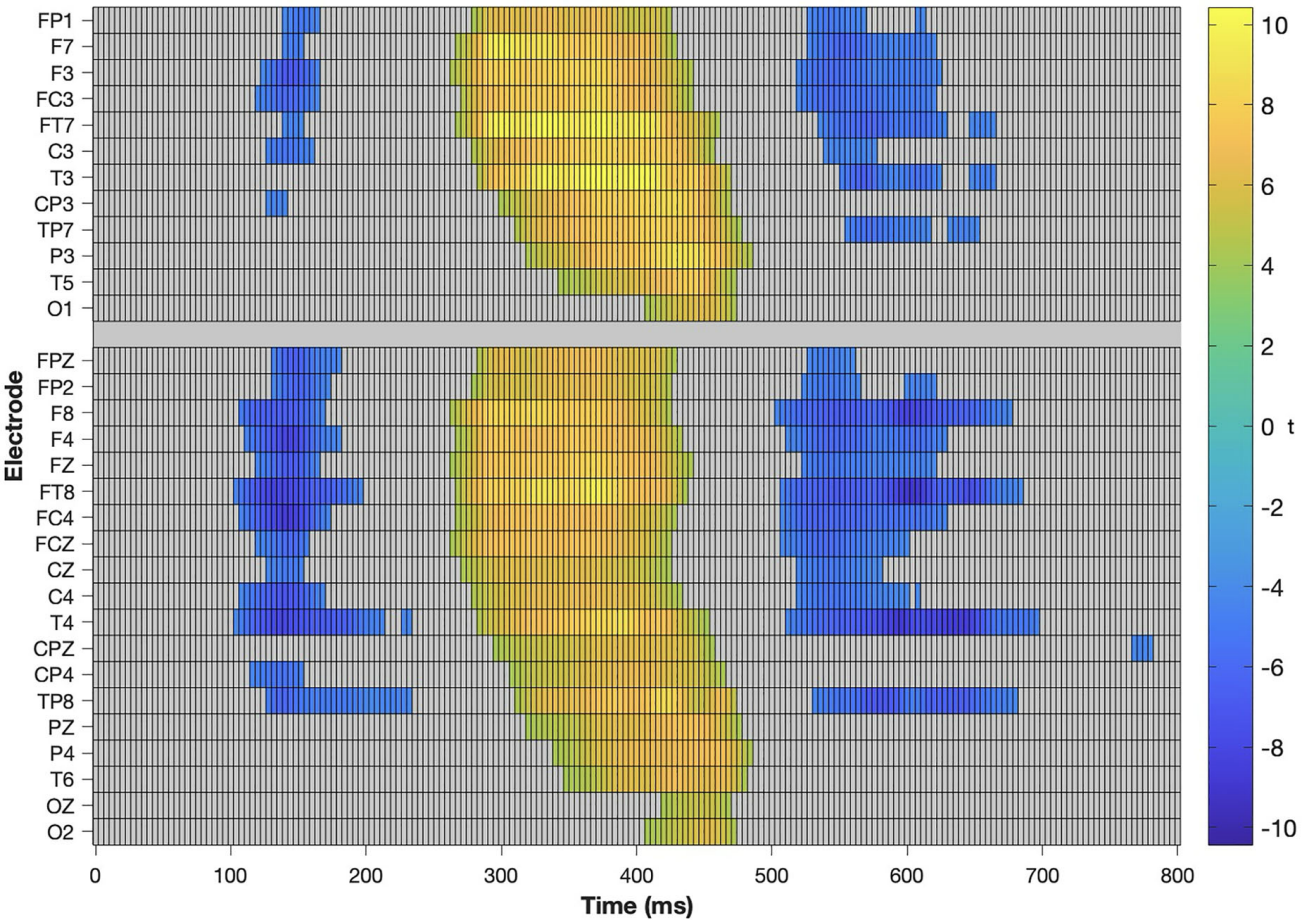
Point‐by‐point comparison of the voltage between the STD and DEV conditions. Only those that exceeded the corrected significance threshold are colored. Cool colors indicate DEV < STD; hot colors indicate STD > DEV.

**FIGURE 6:**
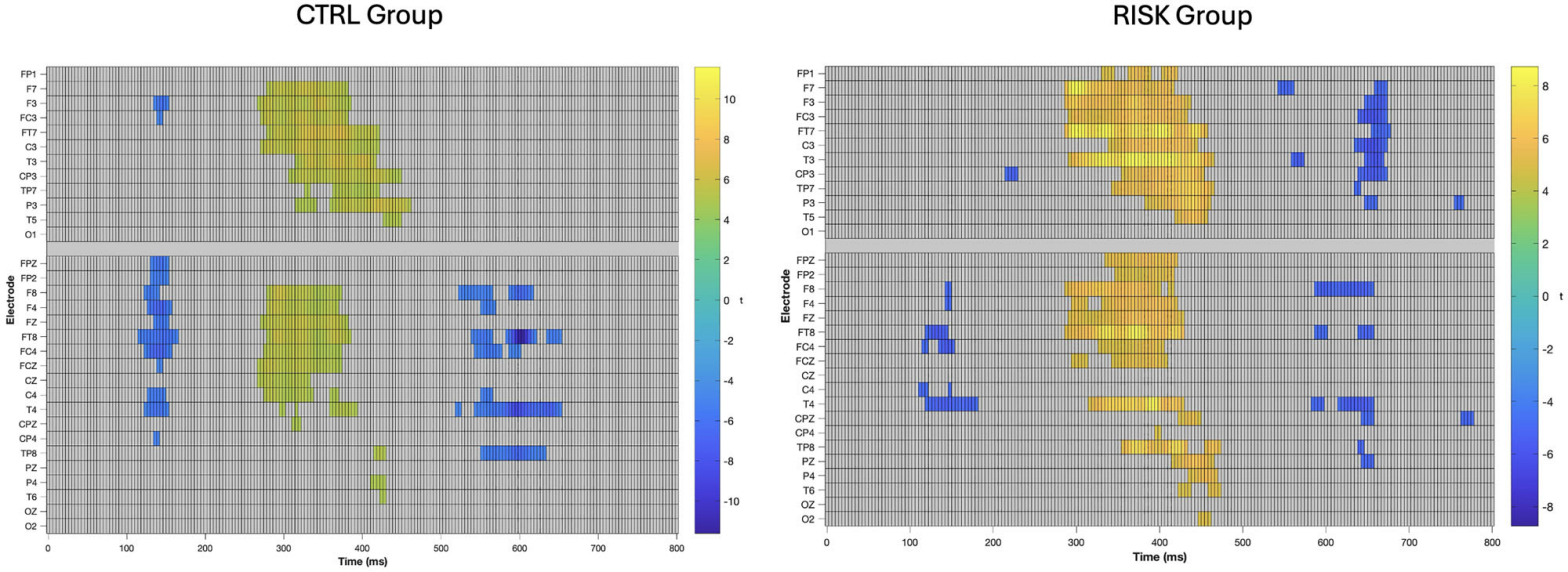
Point‐by‐point comparison of the voltage between the STD and DEV conditions for the CTRL group (*n* = 25, left) and the RISK group (*n* = 25, right). Only those comparisons that exceeded the corrected significance threshold are colored. Cool colors indicate DEV < STD; hot colors indicate STD > DEV.

**FIGURE 7:**
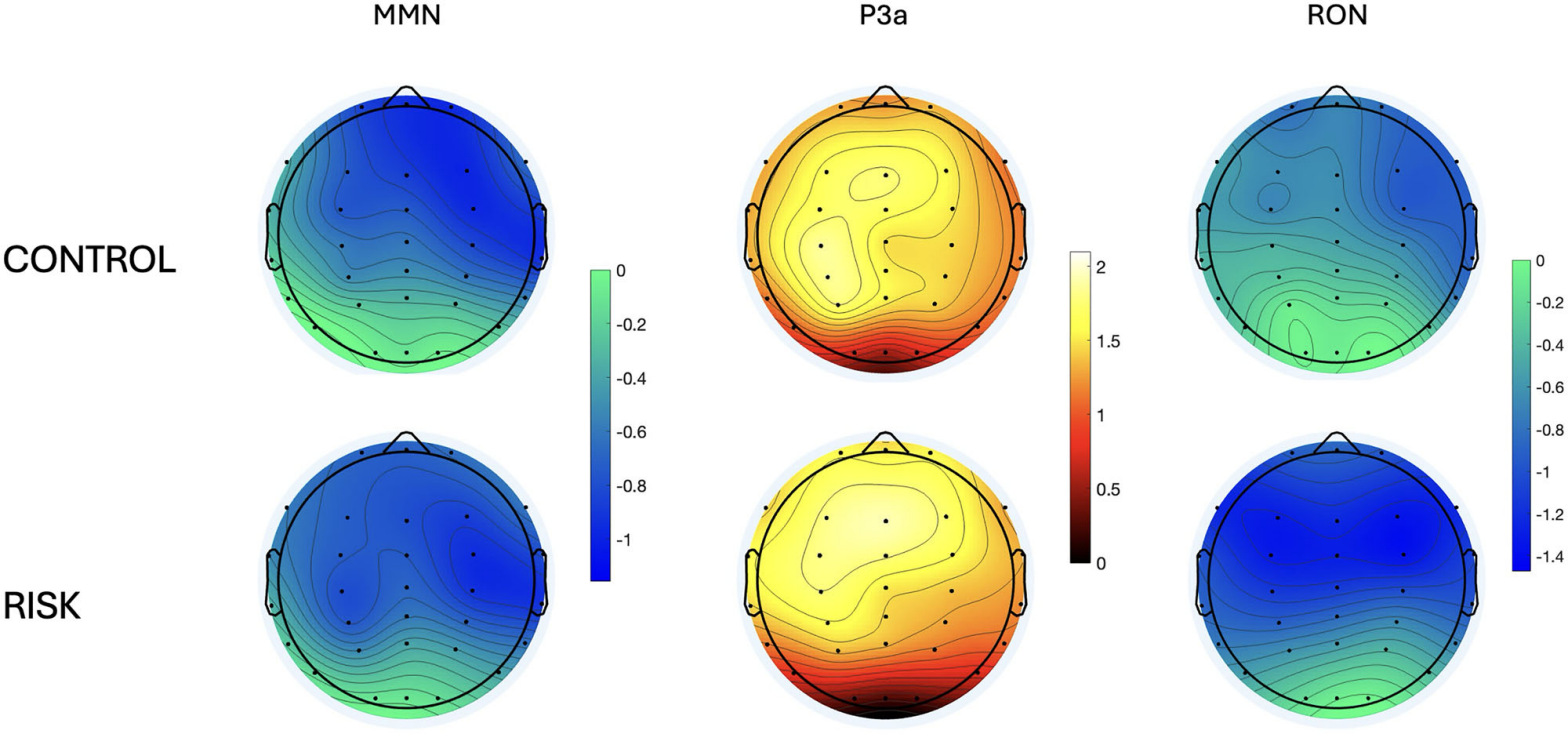
Topographic maps of the mean amplitude of each component for both groups.

The results of the point‐by‐point analyses across the defined epoch at all 32 electrodes are shown in Figure 5. Only the time points that exceeded the critical *t*‐values (i.e., where there was a significant effect of the condition [*p* < 0.05, corrected]) are colored. These results indicated an effect of the stimulus type over the time windows that correspond to the MMN (approximately 100–200 ms), the P3a (approximately 270–480 ms), and the RON (approximately 500–700 ms). The P3a effect was widely distributed across the scalp in both groups, while the MMN and the RON effects were more prominent over the right hemisphere (Figure 6). This difference is also appreciated in the scalp plots shown in Figure 7. We also observed a RON effect on the left side of the RG, which was not present on the CG. This difference was confirmed by a significant interaction effect Group × Laterality in the 3‐way ANOVA conducted on the mean amplitude of the components (*F*_(1.58,76.01)_ = 3.61, *p* = 0.04, *η* ^2^ *_pb_* = 0.07). Post‐hoc tests revealed that the mean amplitude of the component was marginally larger in the left hemisphere for the RG than the CG (*t_(_*_48)_ = 2.00, *p* = 0.0506, *d* = 0.5).

#### 2.2.3 Latencies

**FIGURE 8:**
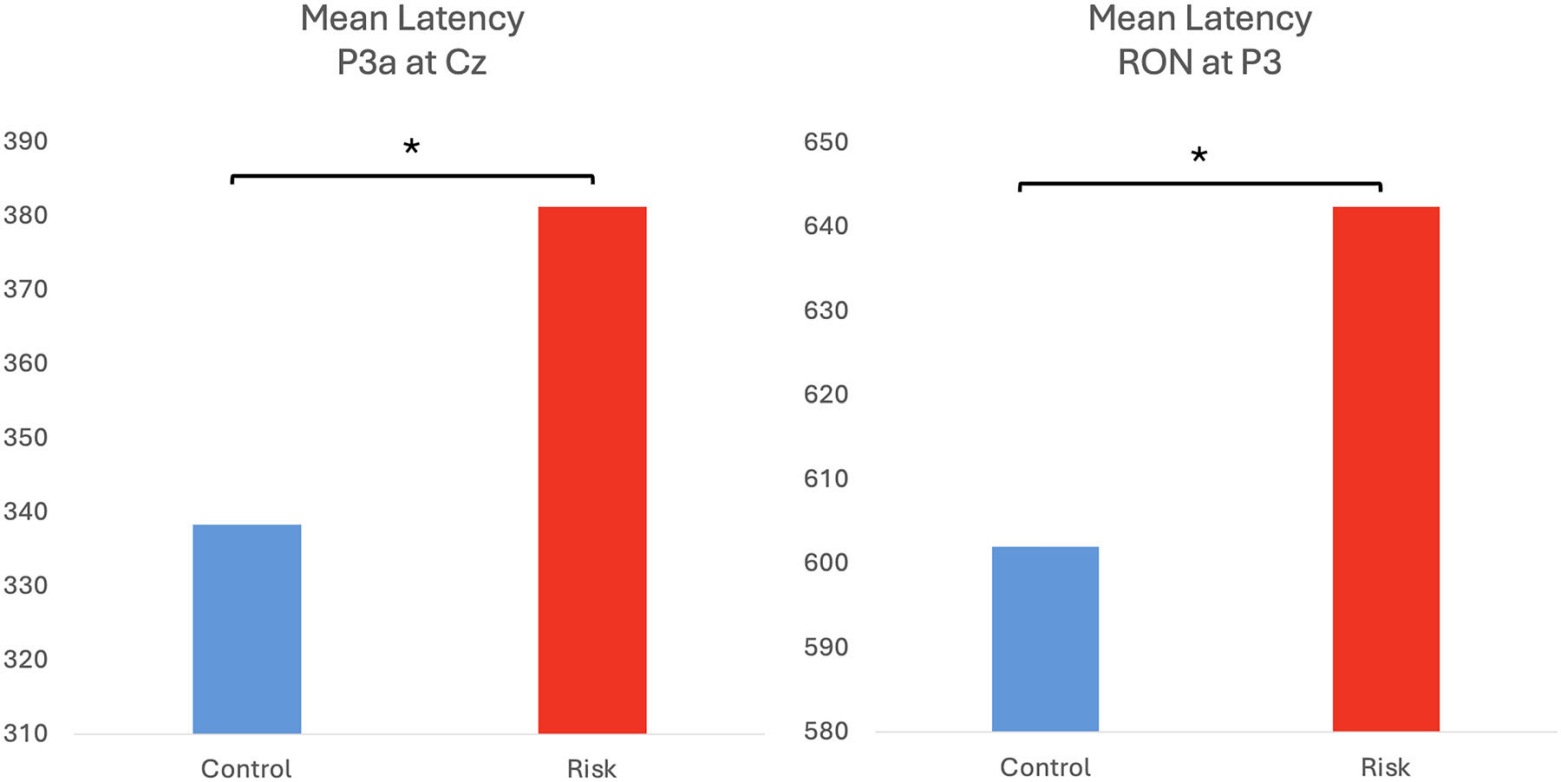
Mean latencies by group for the P3a component at Cz (left) and the RON component at P3 (right). *p < 0.05, corrected for multiple comparisons.

There was no significant group effect on the latency of the MMN component. However, we did find a significantly longer latency of the P3a on the RG over Cz (*t*_(48 _= −2.82, *p* = 0.02, *d* = 0.79) and Pz (*t*_(48)_ = −2.39, *p* = 0.02, *d* = 0.68) as well as that of the RON on the RG over P3 (*t*_(48)_ = −2.49, *p* = 0.045, *d* = 0.71) (Figure 8).

## 3 Discussion

This study aimed to explore whether there are involuntary attention differences between two groups of healthy older adults—one at risk of cognitive impairment—as revealed by the distraction potential.

A point‐by‐point analysis revealed no significant differences between groups in the amplitude of any of the three components of the distraction potential. However, there were differences in the components' latencies and the topographic distribution of the RON effect. While the effect was significant in the right hemisphere for both groups, it was also present in the left hemisphere for the RG. These differences contrast with the absence of behavioral differences between groups in response time and accuracy.

The MMN has been associated with the automatic detection of changes in the auditory context. Regarding its implications for the aging process, previous studies conducted with older adults who have and do not have a clinical diagnosis of cognitive impairment have shown inconsistent results. On one hand, Buranelli et al. (2009) found no significant differences in the amplitude or latency of the MMN between older and young adults. Solís‐Vivanco et al. (2011) found no differences in the amplitude or latency of the MMN between healthy patients and those with Parkinson's disease. Similarly, Papadaniil et al. (2016) did not find differences in the MMN between healthy older adults and those with mild cognitive impairment; however, they did find differences between these two groups and those with AD, who exhibited a lower amplitude of the MMN component. These findings have also been reported in other studies that show changes in the MMN only when the disease‐related impairment is more severe (Pekkonen et al. 2000, 1995). Other studies, however, have found a direct relationship between MMN latency and the progression of neurodegenerative diseases (Gao et al. 2018; Tsolaki et al. 2017). Considering the current results, there does not appear to be a difference in the temporal aspect of the detection of unexpected changes in the physical characteristics of the stimuli between the groups, which points to the maintained integrity of the perceptual process.

The P3a is considered an electrophysiological correlate of the orienting response, and relationships have been found between its amplitude and performance on executive tests, such as the Wisconsin Card Sorting Task (Tsuchiya et al. 2000). In the case of this component, we observed differences involving a longer latency in the at‐RG. In patients with MCI and AD, smaller amplitudes and longer latencies of the P3a wave have been reported compared to healthy controls (Papadaniil et al. 2016). A meta‐analysis conducted by Howe et al. (2014) reported a greater difference in terms of P3a in frontal derivations when studying patients with MCI and AD compared to healthy subjects, where the latency of these components was reportedly proportional to the progression of the cognitive decline. In other words, patients with MCI exhibited longer latencies than the control subjects but shorter latencies than those with AD. Additionally, they found a more posterior distribution of this component in the AD group compared to the other two groups. Although participants in the RG do not exhibit clinical symptoms of cognitive impairment, we observed a delay in the latency of the P3a component in centroparietal midline regions compared to the control group. Considering that these subjects, who display a predictor for cognitive impairment, lie halfway on the continuum between healthy individuals and those with cognitive impairment, this result aligns with previous findings. Specifically, here we report that there is already a delay in involuntary attentional orientation, as reflected by the latency delay of the P3a.

Finally, we also observed differences between groups in the latency and topographic distribution of the RON, which has been associated with redirecting attention towards the stimuli's characteristics relevant to the ongoing task, in this case, the tone duration. The at‐RG exhibited longer latencies than the control group in left parietal areas, which could indicate a delay in the process of reorienting attention or a carryover effect from the delay of previous processes. The latter option seems improbable since the difference was observed on electrodes other than those that resulted in significance for the P3a analysis.

Our findings suggest a significant relationship between the latency of the distraction potential components and the presence of excessive theta activity. This relationship could have implications for our understanding of attentional processes and cognitive function. While a delay in the neural processes involved in the attentional orientation and reorientation was observed in the RG, this delay does not appear sufficiently pronounced to affect behavioral performance, at least for the time being. Conversely, the broader spatial distribution of the RON component effect in the RG may reflect the recruitment of additional brain regions, which we hypothesize to be acting as compensatory mechanisms. These mechanisms could enable these individuals to perform adequately—at least comparably to the control group. Notably, the effect of excessive theta activity on distractibility appears to manifest in attentive processes, as reflected in the P3a and RON component differences, but not in the pre‐attentive ones, such as those indexed by the MMN.

It is essential to acknowledge a potential limitation of the present study, which is the high socioeconomic and educational levels of the participants, thereby reducing the generalizability of our results to the broader population. On the other hand, the strict requirements to fulfill the inclusion criteria give us more certainty that the observed differences between the groups are due to their electroencephalographic characteristics and not to other potentially confounding variables, which are rarely taken into consideration in studies involving older adults. Future studies should confirm the relationship explored in this study and investigate the connection between this potential and performance in other cognitive domains, such as executive functions, in older adults.

In conclusion, we found a delayed neurophysiological response of involuntary attention in healthy adults with an electroencephalographic risk of cognitive impairment. Whether the delay in the distraction potential may predict a future worsening of the cognitive status and the onset of an NCD in this population remains to be explored. In light of these findings and prior results from our research group (Sigg et al. 2025; Sánchez‐Moguel et al. 2018), as well as the evidence reported by Prichep et al. (2006), we note that behavioral assessments alone, although essential and widely used in clinical and research settings, may sometimes fail to differentiate between subjects with subtle subclinical alterations. This limitation may lead to the erroneous assumption that all individuals constitute a homogeneous group of healthy older adults. Behavioral testing, typically relying on neuropsychological batteries, remains the most common practice for establishing control groups or normative databases. However, our and others’ findings suggest that such assessments may inadvertently include individuals with functional alterations that are behaviorally undetectable due to compensatory mechanisms but could manifest clinically within a short timeframe if left unaddressed. Beyond the challenges of establishing reliable control groups or databases, the most critical implication lies in the failure to adopt the EEG as a tool for identifying a biomarker of risk for cognitive impairment in clinical practice. Its implementation will lead to the development of early intervention strategies to prevent or at least slow cognitive decline.

## Author Contributions

**Mauricio González‐López**: conceptualization, methodology, investigation, software, formal analysis, data curation, writing – original draft, writing – review and editing, visualization. **Rodolfo Solís‐Vivanco**: methodology, investigation, writing – review and editing, visualization. **Thalía Harmony**: methodology, investigation, writing – review and editing, visualization. **Thalía Fernández**: conceptualization, methodology, investigation, software, formal analysis, resources, supervision, project administration, funding acquisition, writing – review and editing, visualization.

## Ethics Statement

This study was reviewed and approved by the Bioethics Committee of the Institute of Neurobiology of the National Autonomous University of Mexico (INEU/SA/CB/109).

## Consent

The participants provided their written informed consent to participate in this study.

## Conflicts of Interest

The authors declare no conflicts of interest.

## Peer Review

The peer review history for this article is available at https://publons.com/publon/10.1002/brb3.70970.

## Data Availability

The data that support the findings of this study are available from the corresponding author upon reasonable request.
